# Sensory adaptation for timing perception

**DOI:** 10.1098/rspb.2014.2833

**Published:** 2015-04-22

**Authors:** Warrick Roseboom, Daniel Linares, Shin'ya Nishida

**Affiliations:** 1 NTT Communication Science Laboratories, Nippon Telegraph and Telephone Corporation, 3-1 Morino-sato Wakamiya, Atsugi-shi, Kanagawa, 243-0198, Japan; 2 Department of Basic Psychology, Faculty of Psychology, University of Barcelona, Passeig Vall d'Hebron, 171, 08035 Barcelona, Spain

**Keywords:** recalibration, repulsion, adaptation, vision, multisensory, psychophysics

## Abstract

Recent sensory experience modifies subjective timing perception. For example, when visual events repeatedly lead auditory events, such as when the sound and video tracks of a movie are out of sync, subsequent vision-leads-audio presentations are reported as more simultaneous. This phenomenon could provide insights into the fundamental problem of how timing is represented in the brain, but the underlying mechanisms are poorly understood. Here, we show that the effect of recent experience on timing perception is not just subjective; recent sensory experience also modifies relative timing discrimination. This result indicates that recent sensory history alters the encoding of relative timing in sensory areas, excluding explanations of the subjective phenomenon based only on decision-level changes. The pattern of changes in timing discrimination suggests the existence of two sensory components, similar to those previously reported for visual spatial attributes: a lateral shift in the nonlinear transducer that maps relative timing into perceptual relative timing and an increase in transducer slope around the exposed timing. The existence of these components would suggest that previous explanations of how recent experience may change the sensory encoding of timing, such as changes in sensory latencies or simple implementations of neural population codes, cannot account for the effect of sensory adaptation on timing perception.

## 1. Introduction

Our impression of how related two events are and whether they occurred simultaneously depends on the recent history of their relative timing [1–6]. For example, when a visual event repeatedly leads an auditory event by some interval (e.g. approx. 200 ms), subsequent presentations in which the visual event leads the auditory event are reported as more related and simultaneous [3]. Equivalent changes occur after repeated exposure to an auditory event leading a visual event. Subjective simultaneity is similarly modified by exposure to different combinations of audio, visual and tactile events [7–10]; combinations within a single sensory modality [1,2,6] and combinations of self-generated motor actions with sensory stimuli [5], indicating that the changes in subjective simultaneity induced by the recent history of relative timing are a general rule in time perception.

Understanding the mechanisms by which exposure to relative timing affects judgements of simultaneity might help in resolving the question of how the brain represents relative timing. The mechanisms, however, are poorly understood. One proposal is that after exposure to asynchronous events, subjective asynchrony is reduced, because the sensory latency of one of the events—the time that the brain needs to process an event—changes to become more similar to the sensory latency of the other event, so that the transmission time of the signals within the environment plus the sensory latency is similar for both events [10,11]. This proposal emphasizes a link between subjective relative timing and the sensory latency of the events. Changes to the sensory latency of events are conceptually similar to actively synchronizing the timing between two clocks. According to another proposal, relative timing is encoded by a population of neurons that respond selectively to a limited range of asynchronies. In this case, it is proposed that changes in subjective simultaneity following exposure to a fixed relative timing occur, because the neurons that respond most strongly to that timing reduce their activity [12]. These two proposals support the idea that exposure to relative timing changes the encoding of relative timing in neural circuits dedicated to sensory processing. However, there is little neurophysiological evidence for the existence of these changes. An alternative proposal is that changes in subjective simultaneity are not caused by changes in how relative timing is encoded but by changes in the categorization of events in neural circuits involved in making decisions—a change at a decision rather than at sensory processing level [13]. That is, asynchrony exposure might change the criterion of observers to label the events as simultaneous or not without changing the sensory representation of the events.

In spatial vision, for attributes such as contrast, orientation and motion, the recent history of sensory stimulation has been demonstrated to change the sensory encoding of the stimulus, often leading to changes in its discriminability (see [14–16]). If the recent history of relative timing between two events changes the sensory encoding of its relative timing, then repeated exposure to a fixed relative timing might change not only judgements of appearance, but also observers' performance to discriminate differences in timing between events. By contrast, if changes in subjective simultaneity are a consequence only of altered decisional processes, no changes in performance would be expected [14,16].

To test these possibilities, we measured observers' ability to discriminate synchrony from asynchrony with and without previous repeated exposure to fixed asynchrony or synchrony. We found that exposure to a fixed relative timing changed sensitivity for discriminating asynchrony, indicating that exposure-induced changes in subjective simultaneity are related to changes in the sensory encoding of relative timing. This demonstration of sensory adaptation rules out an account based only on altered decisional processes [13]. The specific changes in relative timing sensitivity that we found suggests the existence of two sensory components: a shift in the nonlinear transducer that maps relative timing into perceptual relative timing and an increase of the slope of the transducer around the exposed relative timing. The increase in transducer slope is inconsistent with a hypothesis based only on changed sensory-latency [10,11], whereas the lateral shift is inconsistent with the previously proposed population code model of asynchrony-tuned neurons [12]. Remarkably, the two sensory components that we found are similar to components previously reported for visual spatial attributes, suggesting that similar mechanisms of sensory coding and adaptation underlie time and space.

## 2. Results

### (a) Exposure to asynchrony/synchrony changes sensitivity

**Figure 1.**
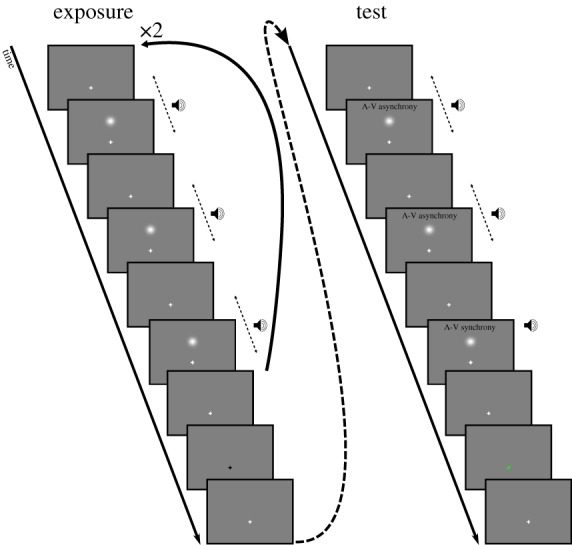
Depiction of example exposure and test sequences. No exposure trials consisted of only the test portion. Exposure sequences consisted of six multisensory pairs with the exception of the sequence presented on the first and the middle trial of each block in which 80 multisensory pairs were presented. The end of the exposure sequence was signalled by the fixation turning black. Feedback (green fixation for correct, red for incorrect) was given to participants.

Three multisensory pairs were presented sequentially; one pair in which the audio (A) and the visual (V) events were in synchrony and two in which the events were asynchronous. The asynchrony between the events was the same for each asynchronous pair and changed on each trial according to the method of constant stimuli [17]. The presentation order of pairs was randomized, and participants needed to identify which pair was different (figure 1). In comparison with the more standard two-interval forced choice task, in this three-interval forced choice task, participants do not need to know in which aspect the pairs differ [17], so it was not necessary to instruct the participants to identify the synchronous (or asynchronous) pair.

**Figure 2.**
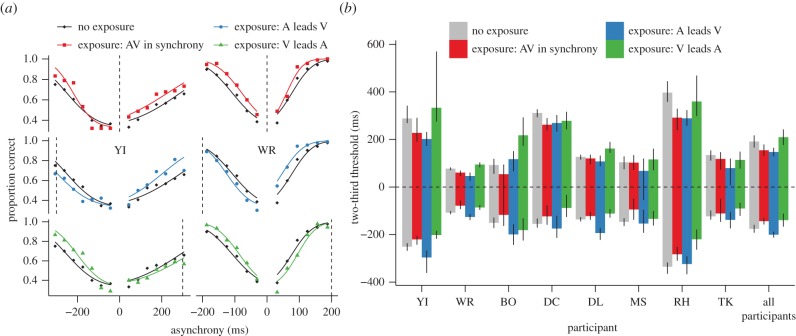
Changes in sensitivity after exposure to asynchrony/synchrony. (*a*) Proportion of correct responses as a function of the asynchrony for participants YI (left) and WR (right) for the different conditions. The dotted vertical lines indicate the exposed asynchrony. For the no exposure condition, the data points and the curves are plotted three times for each participant to facilitate comparison with the other exposure conditions. (*b*) Two-thirds thresholds for each participant and for the average across participants for the different conditions. The error bars correspond to the 95% CIs calculated according to Morey [18].

First, we describe the results for the participants who completed the largest number of trials (YI completed 11 820 trials and WR 11 880 trials, each taking approximately 35 h, see the electronic supplementary material, Methods). As expected, the proportion of correct identifications increased with asynchrony (figure 2*a*, black diamonds). Positive asynchronies indicate audio–visual pairs wherein the visual event leads the auditory event (VA asynchrony) and negative asynchronies indicate that the auditory event leads the visual event (AV asynchrony). Repeated exposure to VA asynchrony impaired the ability of participants to discriminate VA asynchronies, but improved their ability to discriminate AV asynchronies (figure 2*a*, bottom row, green triangles). Similarly, repeated exposure to AV asynchrony impaired discriminability of AV asynchronies, but improved discriminability of VA asynchronies (figure 2*a*, central row, blue circles). Lastly, repeated exposure to audio–visual synchrony improved discriminability of both VA and AV asynchronies (figure 2*a*, top row, red squares).

As a metric of sensitivity for discriminating synchrony from asynchrony for each participant, condition of exposure (VA exposure, AV exposure, synchrony exposure and no exposure) and sign of asynchrony (positive or negative), we fitted a cumulative normal curve (figure 2*a*) and obtained the threshold asynchrony for which the proportion of correct responses was two-thirds (participants WR and YI in figure 2*b*). Relative to thresholds in the no exposure condition, exposure to VA asynchrony increased VA thresholds, but decreased the AV thresholds. Similarly, exposure to AV asynchrony increased AV thresholds, but decreased VA thresholds. Lastly, synchrony exposure decreased both VA and AV thresholds. All differences in thresholds were statistically significant (the 95% bootstrap confidence intervals of the difference was different from zero, see the electronic supplementary material, Methods) excepting the VA threshold for participant YI following exposure to VA asynchrony. However, even in this case, six of the seven data points obtained following exposure to VA asynchronies fall below those for the no exposure condition.

Figure 2*b* shows the thresholds for all participants and their average. A repeated-measures ANOVA for the absolute values of the thresholds with the asynchrony signs (positive or negative) of the four exposure conditions as within-subject factors revealed a significant main effect of exposure (*F*_3,21_ = 3.408, *p* = 0.036, partial *η*^2^ = 0.327) and an interaction between exposure and sign (*F*_3,21_ = 25.016, *p* < 0.001, partial *η*^2^ = 0.781). Comparing no exposure thresholds with those following exposure to AV asynchronies revealed a significant interaction of exposure and sign (*F*_1,7_ = 33.883, *p* = 0.001, partial *η*^2^ = 0.829) such that AV thresholds increased following exposure, whereas VA thresholds decreased. Thresholds following exposure to VA asynchronies mirrored those in relation to the no exposure condition revealing a significant interaction of exposure condition and threshold sign (*F*_1,7_ = 23.424, *p* = 0.002, partial *η*^2^ = 0.77). Comparison of no exposure thresholds with thresholds following synchrony exposure revealed a significant effect (*F*_1,7_ = 20.067, *p* = 0.003, partial *η*^2^ = 0.741) such that both VA and AV thresholds were significantly decreased. This pattern of results is consistent with the individual results for participants WR and YI.

To minimize the impact of order effects such as learning [19], the data from the no exposure condition was collected intermingled with the data from the different exposure conditions. But, indeed, the data from the no exposure condition do not evidence order effects: the overall performance during the first 25% of trials (63% correct) was not significantly different from the average threshold sensitivity during the last 25% of trials (66% correct; *t*_7_ = 1.13, *p* = 0.29).

The changes in sensitivity that we found support the existence of sensory adaptation for relative timing, but what mechanisms produce the pattern of improvements and impairments is unclear. To address this issue, we considered how adaptation affects perception of visual spatial attributes, such as contrast, orientation or motion. For these attributes, the mechanisms of adaptation have been extensively studied [14–16,20] and, in terms of how adaptation changes transduction of the attribute, can be broadly differentiated into two descriptive categories: a lateral shift and repulsion [14,16]. Next, we considered whether a lateral shift or/and repulsion can explain the sensitivity changes that we found.

### (b) Lateral shift

**Figure 3.**
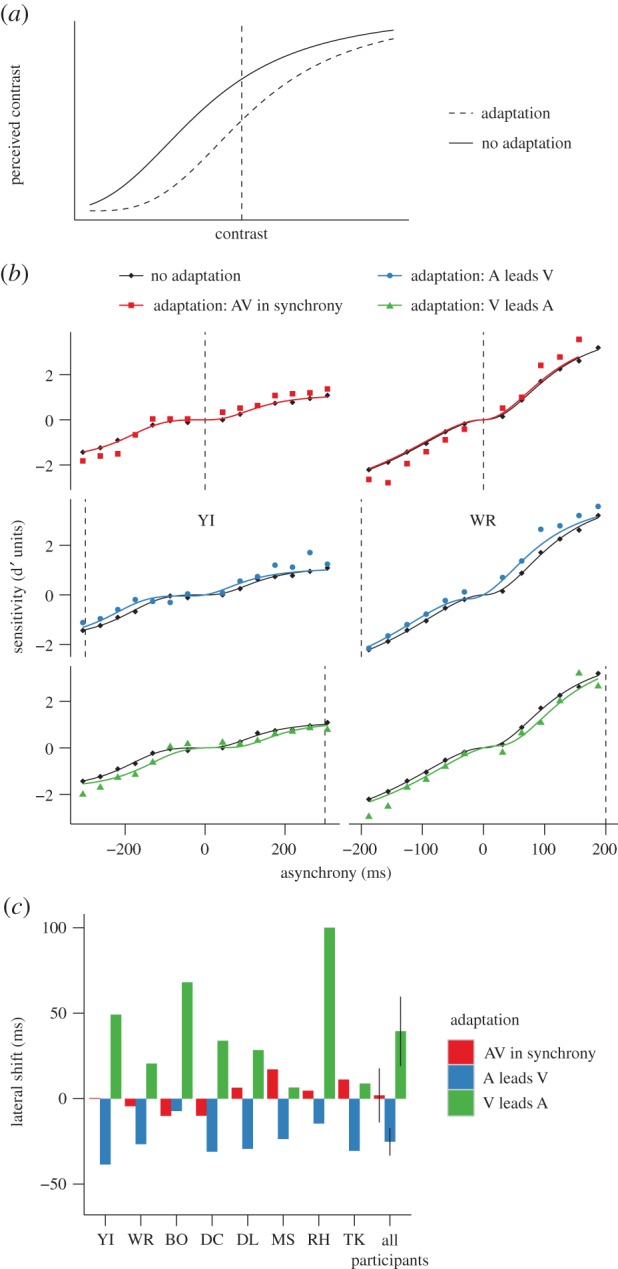
A lateral shift in transducer. (*a*) Lateral shift of contrast. (*b*) Sensitivity as a function of the asynchrony for participants YI (left) and WR (right) for the different conditions. These data are the same as plotted in figure 2. The black curves correspond to the transducers in equation (2.1) that best fitted the data for the no adaptation condition. The coloured curves correspond to the laterally shifted curves that best fitted the data for the different adaptation conditions. For the no adaptation condition, the data points and the curves are plotted three times for each participant to facilitate comparison with the other adaptation conditions. (*c*) Best lateral shift parameter for each participant and average across participants for the different adaptation conditions. The error bars correspond to the within-subject 95% CIs calculated according to Morey [18].

A ‘lateral shift’ describes a shift towards the exposed (adapted) value of the function that transduces the physical magnitude of the attribute into the corresponding perceptual space. Figure 3*a*, for example, shows how adaptation to high-contrast shifts the transduction of contrast towards higher contrast levels, which would also produce changes at the adaptor [14,21]. A lateral shift can explain (under the assumption of additive noise, see Discussion) a change in discriminability when transduction of a physical attribute (physical relative timing in this case) to a perceptual response (perceptual relative timing) is nonlinear [21–23].

To assess whether the adaptation-induced changes in sensitivity that we found can be explained by a lateral shift, we first estimated how physical relative timings are transduced into perceptual relative timings using the data from the no adaptation condition. To do this, we assumed that equally discriminable pairs of physical intensities produce the same difference in magnitude of the perceptual response which, under the framework of signal detection theory, corresponds to assuming that the perceptual response is affected by additive noise and can be effectively implemented by a transformation of the proportion of correct responses into dprime units [17,22,24,25]. We transformed the proportion of correct responses in figure 2*a* into dprime units using standard procedures (see the electronic supplementary material, Methods) and reversed the sign of dprime for AV asynchronies to depict a transducer that maps VA and AV asynchronies into positive and negative perceptual space (black diamonds in figure 3*b*). For each participant, condition of adaptation and sign of asynchrony (positive or negative), we fitted the dprime measures using the following three-parameter transducer2.1
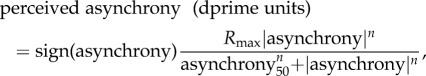
where *R*_max_ corresponds to the maximum response, asynchrony_50_ corresponds to the asynchrony for which the response is half of the maximum response and *n* determines the steepness of the function. Consistent with previous findings [23], we found that for most participants the transducer has an expansive nonlinearity at small VA and AV asynchronies indicating worse discrimination within that region (pedestal effect; black curves in figure 3*b* shows the transducers for two participants—plotted three times to facilitate the comparison with the adaptation conditions).

For each adaptation condition, we calculated dprime (coloured points in figure 3*b*) and modelled the lateral shift with a single parameter corresponding to a horizontal displacement of the non-adapted transducers. We then calculated sensitivity at each asynchrony by subtracting the value of the shifted transducer at 0 ms asynchrony from the value of the shifted transducer at that asynchrony (coloured curves in figure 3*b*).

To evaluate how the lateral shift model fitted sensitivity after adaptation, we compared the mean squared error (MSE) of ‘adapted’ dprimes (coloured points) using the laterally shifted curves (coloured curves) to the MSE of ‘adapted’ dprimes using the non-adapted transducers (black curves) (electronic supplementary material, figure S1). The model improved the fit for the asynchrony adaptation conditions: MSE was reduced after VA adaptation (paired *t*-test: *t*_7_ = 3.36, *p* = 0.012) and AV adaptation (*t*_7_ = 3.18, *p* = 0.016). Nevertheless, the model did not improve the fit for synchrony adaptation (*t*_7_ = 1.84, *p* = 0.11).

### (c) Repulsion

**Figure 4.**
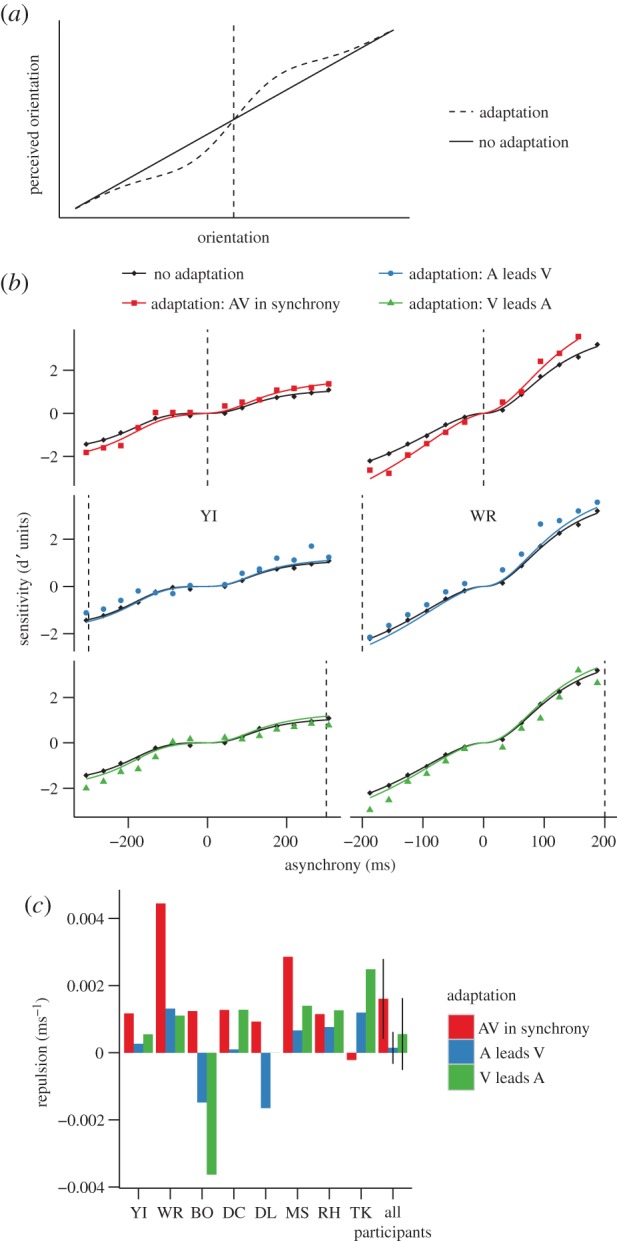
Repulsion. (*a*) Repulsion of orientation. (*b*) Sensitivity as a function of the asynchrony for participants YI (left) and WR (right) for the different conditions. These data are the same as plotted in figure 2. The black curves correspond to transducers in equation (2.1) that best fitted the data for the no adaptation condition. The coloured curves correspond to the curves for repulsion that best fitted the data for the different adaptation conditions. (*c*) Best parameter for the repulsion model for each participant and for the average across participants for the different adaptation conditions. The error bars correspond to the within-subjects 95% CIs calculated according to Morey [18].

Repulsion describes a change in the transducer around the adaptor such that the transduced value at the adaptor does not change, whereas values close to the adaptor are ‘repelled’ away from it (figure 4*a* shows a simplified example for the transduction for orientation; [15,20]). A recent study reported that repulsion might contribute to changes in subjective relative timing judgements following adaptation [12].

To assess whether the adaptation-induced changes in sensitivity that we found can be explained by repulsion, we used the same procedure as that for the lateral shift, but instead perturbed the non-adapted transducer around the adaptor using the derivative of a Gaussian function like the one depicted in figure 4*a* (two-parameter model: amplitude and standard deviation of a Gaussian). We found that for 12 of the 24 fits (three adaptation conditions: VA, AV and synchrony × 8 participants) the parameter controlling the extent of the perturbation (standard deviation of the Gaussian) reached the upper bound that we set, indicating that the extent of repulsion was beyond the range of the asynchronies that we tested. For this reason, we approximated repulsion using a single parameter model that corresponded to the slope of a linear function centred on the adaptor and added to the non-adapted transducer, which effectively rotates the transducer clockwise or anti-clockwise around the point of adaptation. We then calculated sensitivity at each asynchrony by subtracting the value of the shifted transducer at asynchrony = 0 from the value of the ‘repulsion’ transducer at that asynchrony (coloured curves in figure 4*b*).

To evaluate how the repulsion model fitted sensitivity after adaptation, we compared the MSE for the repulsion curves and the non-adapted transducers (electronic supplementary material, figure S1). Repulsion improved the fit for the synchrony adaptation condition: MSE was reduced (*t*_7_ = 3.06, *p* = 0.012). Repulsion did not improve the fit for VA adaptation (*t*_7_ = 2.32, *p* = 0.053). Although not apparent for participants YI and WR (figure 4*b*), repulsion also improved the fit for AV adaptation across participants (*t*_7_ = 3.60, *p* = 0.0089). The slope of the model, however, was significantly positive and different from zero—indicating repulsion—only for synchrony adaptation (confidence intervals (CIs) in figure 4*c*). We think that the lack of significant changes in the slope of the transducers for the asynchronous adaptation conditions might be related to less reliable data around the asynchronous adaptors. As we measured discrimination around synchrony—far from the asynchronous adaptors—the proportion of correct responses near the asynchronous adaptors is close to 1 and thus less informative.

### (d) Lateral shift plus repulsion

**Figure 5.**
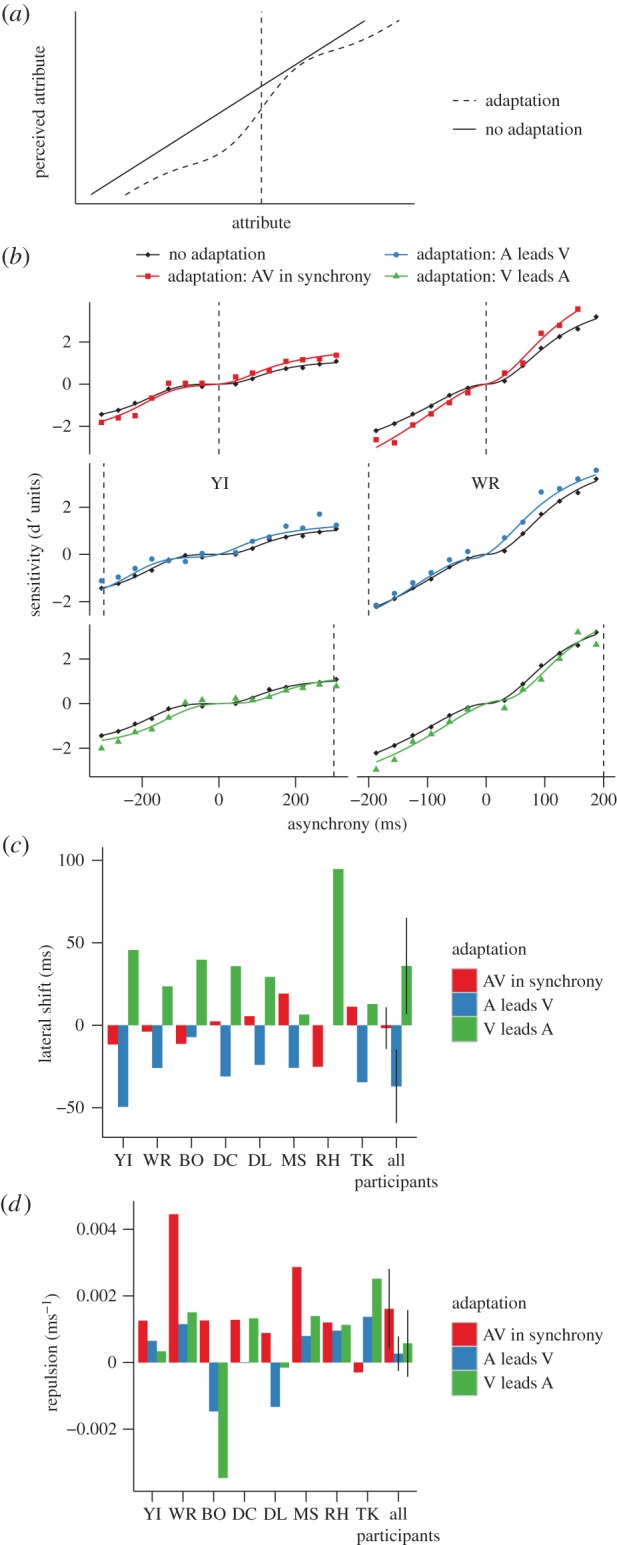
Lateral shift plus repulsion. (*a*) Depiction of the model. (*b*) Sensitivity as a function of the asynchrony for participants YI (left) and WR (right) for the different conditions. These data are the same as plotted in figure 2. The black curves correspond to transducers in equation (2.1) that best fitted the data for the no adaptation condition. The coloured curves correspond to the curves for lateral shift-plus-repulsion that best fitted the data for the different adaptation conditions. (*c*) Best parameter for the lateral shift-plus-repulsion model for each participant and for the average across participants for the different adaptation conditions. The error bars correspond to the within-subjects 95% CIs calculated according to Morey [18].

We assessed whether the combination of the two previous models (figure 5*a,b*) provided a better fit of the changes in sensitivity caused by adaptation (electronic supplementary material, figure S1). The lateral-shift-plus-repulsion model in comparison with the lateral shift model improved the fit for all conditions: VA adaptation (*t*_7_ = 2.47, *p* = 0.042); AV adaptation (*t*_7_ = 3.63, *p* = 0.0084) and synchrony adaptation (*t*_7_ = 3.042, *p* = 0.019). The lateral-shift-plus-repulsion model in comparison with the repulsion model improved the fit for the asynchrony adaptation conditions: VA adaptation (*t*_7_ = 4.05, *p* = 0.0049) and AV adaptation (*t*_7_ = 3.50, *p* = 0.010). It did not improve the fit for the synchrony adaptation condition (*t*_7_ = 1.71, *p* = 0.13). The improvements were not just owing to an increase in the number of the free parameters as, when combining all conditions, the Akaike information criterion (see the electronic supplementary material, Methods) for all participants was lower for the lateral shift-plus-repulsion model indicating that it is a more probable model (see the electronic supplementary material, figure S1). Confirming the superiority of the lateral shift-plus-repulsion model, likelihood ratio tests (see the electronic supplementary material, Methods) for the lateral shift and repulsion models relative to the lateral shift-plus-repulsion were highly significant for all participants (the largest probability was for participant BO for the comparison of repulsion versus lateral shift-plus-repulsion: *p* = 0.02, *χ*_1_ = 5.41). As for the two parameters of the combined model, they were very similar to the parameters obtained by independent fits of lateral shift and repulsion (figure 5*c,d*).

## 3. Discussion

We found that repeated exposure to positive asynchronies impaired discrimination of positive asynchronies, but improved discrimination of negative asynchronies. We found equivalent changes following exposure to negative asynchronies. Furthermore, exposure to synchrony improved the discriminability of both positive and negative asynchronies. These changes in discriminability provide, to our knowledge, the first direct evidence that exposure to temporal relationships causes sensory adaptation. Consequently, our results explicitly rule out the possibility that the changes in subjective simultaneity found in previous studies [3–6,26] can be explained by only high-level decisional factors [13].

### (a) Descriptive mechanisms: changes in the transducers

To understand the pattern of sensitivity changes that we found, we fitted the data with three descriptive models inspired by how adaptation affects the transduction of visual spatial attributes. The first model assumes only a *lateral shift* in the nonlinear transducer that maps relative timing into perceptual relative timing. The model did a fairly good job in predicting the performance changes after asynchrony adaptation. Furthermore, given that the transducer for relative timing is approximately reflected around the zero-point (figures 3–5; see also figure 2*c,f*, [23]), the shift towards the adaptor not only changed sensitivity nearby the adaptor, but also far from it—a characteristic that parallels the effects of adaptation for visual spatial attributes [27–29].

The lateral shift predicted for each participant the direction of the effect of adaptation that previous studies [3–10] have found when directly measuring the shift in subjective simultaneity (figure 3*c*). Across participants, the shift was positive after adaptation to a VA asynchrony, negative after adaptation to an AV asynchrony and no different from zero for synchrony adaptation (figure 3*c*). Remarkably, the magnitude of shift that we found is similar to the shift in subjective simultaneity measured in previous studies [3]. These results are not obvious given that we did not measure appearance but performance. If, for example, the discrimination of temporal intervals were independent of the length of the interval—e.g. linear transduction (coupled with additive noise, see below)—then it would be impossible to recover the magnitude of the shift from performance data, as discrimination before and following adaptation would be no different regardless of changes in subjective simultaneity.

The lateral shift did not describe well the improvements in sensitivity around synchrony following synchrony adaptation. We found, however, that these changes were well explained by a *repulsion* model conceptually similar to that proposed by Roach *et al*. consisting of an increase of the slope of the transducer around synchrony. The repulsion model also provided a reasonable fit of the changes in sensitivity after negative (AV) adaptation, consistent with that previously reported [12], but not after positive adaptation (VA). We can only speculate about the underlying reason for this asymmetry, but asymmetries in the perception of AV and VA asynchronies have been reported before [19,30,31].

The lateral shift and repulsion models by themselves could each capture part of the changes in sensitivity, but could not explain the whole pattern of results. Our modelling indicates that a *lateral shift followed by repulsion*—a combination that to the best of our knowledge has not been used before to explain adaptation of any sensory attribute—is needed to describe how adaptation to temporal relationships changes sensitivity to discriminate synchrony. This indicates that our results not only rule out decisional factors to explain exposure-induced changes in subjective timing, but are also inconsistent with the two previously suggested implementations of sensory adaptation for temporal relationships: changes in the sensory latency of the events [10,11] and a simple population coding model in which exposure changes the response gain of neurons tuned to asynchrony [12].

One limitation of our modelling of performance is that we estimated the transducers assuming that the sensory noise was additive and did not change with adaptation [17,22,24,25], making discriminability directly related to the shape of the transducers [24,25]. Another extreme possibility is that adaptation does not influence the shape of the transducer, but changes the noise. In this case, however, the changes in discriminability would not be accompanied by changes in subjective simultaneity, which is not consistent with previous findings [3–6]. As there is some evidence that for relative timing exposure does affect the noise [12], a more realistic model possibly should include changes in the transducers and the noise. But, disentangling the effects of a manipulation on the transducers and the noise is quite difficult [32] and might require an extremely time consuming experiment in which asynchrony perception is measured subjectively and objectively at several asynchronies following adaptation to several asynchronies.

### (b) Function

For some visual attributes such as contrast, a lateral shift of the transducer towards the adaptor has been functionally associated with recalibration [14,21]. Recalibration is useful when the range of possible values of the attribute exceeds the possible range of the perceptual representation. Under such circumstances, a shift in the transducer will place the perceptual range closer to the values of the attribute recently presented increasing sensitivity to differences in the attribute [14,16]. For other spatial attributes such as orientation, repulsion, expanding the perceptual range around the adaptor (figure 4*a*), has been also associated with increased sensitivity to recently presented values of the attribute [20,33]. These views of adaptation for spatial attributes suggest that the function of adaptation for time relationships might be to increase sensitivity to recently presented relative timings.

In contrast with the function of enhanced sensitivity around the adaptor described above, the common proposed function of adaptation for timing relationships is that exposure to asynchrony recalibrates the synchrony point to enhance the integration of events [23,31]. For example, enhanced integration for audio–visual speech will increase its intelligibility [34]. As enhanced integration is associated with a decrease in sensitivity [35,36], within this view, enhanced integration caused by asynchrony adaptation should be associated with a decrease in discrimination sensitivity, because it widens the range of asynchronies perceptually indistinguishable from physical synchrony. We did find a decrease in sensitivity for asynchronies around the synchrony point in the direction of the adaptor that was related to the lateral shift of the expansive nonlinearity of the transducer around synchrony. Whether this decrease in sensitivity can be linked to the typically proposed function of enhanced integration remains to be seen.

### (c) Neural mechanisms

Changes in the neural latencies of the events [10,11] could possibly be a simple implementation of the lateral shift of the transducer caused by adaptation, but it is unclear how latency changes might produce repulsion. Population-codes of asynchrony-tuned neurons in which adaptation selectively reduces the response gain of the neurons tuned to the adaptor value could explain repulsion [12], but not the lateral shift. A similar model that includes neurons whose response increases monotonically with asynchrony might explain the lateral shift, but not repulsion [37]. Further investigation is needed to determine whether more complex population-coding models such as models in which neurons that are not selectively responsive to the adaptor nevertheless change their response following adaptation—like those reported for orientation [28]—can explain the lateral shift and repulsion together.

### (d) Conclusion

In this study, we found that repeated exposure to a fixed timing relationship changes performance to discriminate synchrony from asynchrony. This result provides, to the best of our knowledge, the first direct evidence for sensory adaptation in the context of relative timing and rules out exclusively decision-level accounts of the subjective timing effect. Further, our modelling suggests the existence of two sensory components, each inconsistent with one of the previously suggested accounts of sensory change following asynchrony exposure: sensory latency change or a simple implementation of a population code based on asynchrony-tuned neurons. Our results are, however, broadly consistent with those previously reported in the context of spatial vision [27,28], supporting the idea that the mechanisms of neural coding and adaptation are similar for time and space.

## 4. Methods

### (a) Participants

Participants included two of the authors (W.R. and D.L.) and six further participants, five of which were naive as to the experimental purpose. All reported normal or corrected to normal vision and hearing. Informed consent was obtained from all participants.

### (b) Apparatus and stimulus

Visual stimuli were generated using a ViSaGe mark I from Cambridge Research Systems (CRS) and displayed on a 21″ Sony Trinitron GDM-F520 monitor (resolution of 800 × 600 pixels and refresh rate of 160 Hz). Participants viewed stimuli from a distance of approximately 57 cm. Audio signals were presented binaurally via Sennheiser HDA200 headphones. Audio stimulus presentations were controlled by a Tucker-Davis Technologies RM1 mobile processor. Auditory presentation timing was driven via a digital line out from the ViSaGe, which triggered the RM1. Participants responded using a CRS CB6 response box.

Visual events were a luminance modulated Gaussian blob (standard deviation of the blob was 0.7 degrees of visual angle (dva); peak luminance difference from background was approx. 38 cd m^−2^; figure 1) displayed against a grey (approx. 38 cd m^−2^) background. A white (approx. 76 cd m^−2^) fixation crosshair (subtending 0.4 dva) was presented centrally with the blob appearing 1.5 dva above the crosshair. The blob was presented for two consecutive frames, approximating 12.5 ms in duration. Auditory signals were a 12.5 ms amplitude pulse, containing 2 ms cosine onset and offset ramps of 1500 Hz sine-wave carrier at approximately 55 db SPL.

### (c) Procedure

The task used a three-interval odd-one-out method. In one of the three intervals, a physically synchronous audio–visual pair was presented while in the other two intervals physically identical asynchronous audio–visual pairs were presented. Participants needed to select which of the three intervals (first, second or third) contained an audio–visual pair that was different from that presented in other two intervals. Physically asynchronous pairs were pseudo-randomly selected on each trial from a range of possible asynchronous values (e.g. ±37.5 : 187.5 ms in 37.5 ms steps) according to a method of constant stimuli. The range of asynchronous values was tailored for each individual participant based on pre-tests indicating their performance (maximum range of between 187.5 and 437.5 ms). The position of the synchronous presentation in the three intervals was distributed such that in a single block of trials participants completed two trials with the synchronous audio–visual pair presented at each position (first, second or third), for each level of asynchrony. Blocks of trials could consist of between 10 and 14 levels of asynchrony, depending on participant. Consequently, block length varied between 60 and 84 trials. The interval between successive presentations in the test sequence was 500 ms plus a random interval of up to double the length of the maximum asynchrony for that participant (e.g. for an observer where the largest test asynchrony is 187.5 ms, the interval would be a value between 0 and 375 ms; up to approx. 875 ms depending on participant). This manipulation was to match the randomization of inter-presentation interval as a function of the asynchronous test stimuli so that inter-presentation interval could not be used as a cue to the presented audio–visual relationship. Participants were given trial-by-trial feedback indicating whether they had correctly identified the different presentation by the fixation turning green for 500 ms if correct or red if incorrect.

We investigated audio–visual timing sensitivity under four conditions of exposure to audio–visual asynchrony: no exposure, audio-leads-visual, audio–visual physically synchronous and visual-leads-audio. A standard block of no exposure trials consisted only of test sequences as described above. The duration of a no exposure block of trials was approximately 10 min. For each of the other three exposure conditions, participants completed an exposure-test procedure similar to that used in previous studies [3]. At the beginning of a block of exposure trials, participants observed 80 repeats of the fixed timing relationship (audio-leads-vision, audio–visual synchrony or vision-leads-audio). For the asynchronous exposure, as was the case for the range of asynchronous test values, the asynchrony was tailored for each participant based on previous audio–visual timing discrimination performance. The exposure value was designed to be an audio–visual timing relationship at which that participant was clearly able (i.e. approx. 100% of the time on preliminary tests) to distinguish that the presented audio–visual timing relationship was asynchronous (exposure values from 200 to 400 ms).

During exposure, participants were asked to monitor the audio–visual presentations. On a given presentation, there was a 5% chance an oddball stimulus would be presented. Half of these oddball presentations were visual oddballs and half auditory oddballs. When the oddball was visual, the visual stimulus was presented at half size (standard deviation of the Gaussian blob was 0.35 dva). When the oddball was auditory, the auditory stimulus used a 2500 Hz sine-wave carrier tone rather than used the standard 1500 Hz. Participants pressed a button as quickly as possible when these oddballs were presented. This task was to ensure that participants were actively observing the presentations during exposure periods. The inter-presentation interval for exposure stimuli was 200 ms plus a randomized period up to 250 ms.

Following the initial exposure sequence, the experiment entered an exposure-top-up/test phase. Each trial began with an exposure-top-up period of six presentations identical to those presented in the longer exposure phase (except for the middle trial of the block of trials which again contained 80 exposure presentations). The oddball task was also present during exposure-top-up, though only appeared within the first five presentations so that participants were not distracted by the oddball task during the subsequent test presentation. After the exposure-top-up sequence, the fixation would change from white to black (approx. 0 cd m^−2^) for 500 ms to inform participants that the next presentation would begin the test sequence. The test sequence would then proceed as described for the no exposure condition. The average duration of an exposure block of trials was 25–30 min. Participants always completed a given number of blocks of no exposure trials, followed by the same number of blocks of an exposure condition. The order of completion of exposure conditions was counterbalanced across participants. Following the completion of the final exposure condition, participants completed a final set of no exposure trials. Experimental sessions were extremely long, and there were several testing conditions, so it was not possible for all participants to complete the same number of trials. See the electronic supplementary material, Methods for details of each participant's completed trials.

## Supplementary Material

Supplementary_Methods_Final.docx

## Supplementary Material

Sup_Figure_1.pdf

## Supplementary Material

BO.txt

## Supplementary Material

DC.txt

## Supplementary Material

DL.txt

## Supplementary Material

MS.txt

## Supplementary Material

RH.txt

## Supplementary Material

TK.txt

## Supplementary Material

WR.txt

## Supplementary Material

YI.txt
