# Supplementary material for: Sensory adaptation for timing perception
Source: Proc Biol Sci. 2015 Apr 22;282(1805):20142833. doi: 10.1098/rspb.2014.2833 (PMC4389610; doi:10.1098/rspb.2014.2833)
Supplement: Supplementary_Methods_Final.docx [file rspb20142833supp1.docx]

**SUPPLEMENTARY METHODS**

Experimental sessions were extremely long and there were several testing conditions, so it was not possible for all participants to complete the same number of trials. Author WR completed 7560 trials; 4320 following no exposure, 1080 following each of the other three exposure conditions. Participant YI completed 7620 trials; 4200 following no exposure, 1140 following each of the other three exposure conditions. Author DL and participant RH each completed 5460 trials; 2940 following no exposure; 840 following each of the other three exposure conditions. Participant DC completed 5328 trials; 3060 following no exposure, 780 following exposure to audio leads vision, 720 following exposure to audio lags vision, and 768 following exposure to audio-visual synchrony. Participants MS and BO each completed 1872 trials; 1008 following no exposure, 288 following each of the other three exposure conditions. Participant TK completed 1560 trials; 840 following no exposure, 240 following each of the other three exposure conditions. See also the Supplementary Data Files.

**Analysis**

Analyses were conducted and data figures produced using *R* statistical package (R core team, 2013) via *R Studio IDE* (RStudio, 2012). For each participant, condition of exposure, and sign, we fitted — using maximum likelihood estimation (Kingdom & Prins, 2009; Knoblauch & Maloney, 2012; Lu & Dosher, 2013) — the proportion of correct responses as a function of the asynchrony with cumulative normal functions with the mean and standard deviation as free parameters. We fixed the lower asymptote to 1/3 (chance performance for the three-interval task) and the upper asymptote to 1 (no adjustment for lapses).

To statistically assess the differences between these conditions we used bootstrap methods. Using the obtained MLE parameters for the fit in each experimental condition, for each subject, we simulated 10000 samples of the difference between the no exposure condition, and each of the exposure conditions.

**Models**

We transformed the proportion of correct responses—excepting those equal to 100%—into dprime units according to standard procedures for an m alternatives forced choice task. More specifically, we used the function *dprime.mAFC* from the *psyphy* package for R that implements equation 1 from Green and Day (1991), which also corresponds to equation 6.6 in Kingdom and Prins (2009). For each participant, condition of exposure and sign, we fitted —using minimum least squares (Knoblauch & Maloney, 2012; Lu & Dosher, 2013) — the sensitivity to detect asynchronies using the transducers in Equation 1. To obtain the best parameters for the lateral shift, repulsion and lateral shift-plus-repulsion model, we use minimum least squares.

To compare the performance of the three different models and determine whether the lateral shift-plus-repulsion model only performed better than the other two because of the use of an additional parameter, we computed the Akaike Information Criterion (AIC; Burnham & Anderson, 2002) for each model. AIC is a measure of the relative goodness-of-fit of statistical models and is defined as:

$$AIC=2k-2ln(L)$$

where k is the number of free parameters and L is the maximized value of the likelihood function for the model. The preferred model is the one with minimum AIC.

To further compare the models, we also computed the ratios between the likelihoods of the models

*Λ_lateral-shift_ = L _lateral-shift_ / L _lateral-shift-plus-repulsion_*

*Λ_repulsion_ = L _repulsion_ / L _lateral-shift-plus-repulsion_*

and performed likelihood ratio tests (Kingdom & Prins, 2009) using the statistic D = 2 ln (Λ), which is asymptotically χ^2^-distributed with degrees of freedom equal to the difference in the number of parameters of the models.

**REFERENCES**

Burnham KP, Anderson DR (2002). *Model selection and multi-model inference: A practical information-theoretic approach*. 2nd Ed. New York: Springer.

Green DM, Dai H (1991) Probability of being correct with 1 of M orthogonal signals. *Perception & Psychophysics*, 49, 100–101.

Kingdom FA, Prins N (2009) *Psychophysics: A practical introduction*. London: Academic Press.

Knoblauch K (2014) psyphy: Functions for analyzing psychophysical data in R. R package version

0.1-9. <http://CRAN.R-project.org/package=psyphy>

Knoblauch K, Maloney LT (2012) *Modeling Psychophysical Data in R.*New York: Springer.

Lu Z-L, Dosher B (2013) *Visual Psychophysics: From Laboratory to Theory*. MIT Press.

Morey RD (2008) Confidence intervals from normalized data: A correction to Cousineau (2005). *Tutorials in Quantitative Methods for Psychology, 4,* 61–64.

R Core Team (2013) R: A language and environment for statistical computing. R Foundation for Statistical Computing, Vienna, Austria. URL <http://www.R-project.org/>.

RStudio (2012) RStudio: Integrated development environment for R (Version 0.96.122) [Computer software]. Boston, MA. Retrieved May 20, 2012.

Sup Fig. 1. Comparison of the different models. (A) Average MSE across participants for each model and exposure condition. (B). Average AIC for each model. The error bars correspond to the within-subjects 95% confidence intervals calculated according to Morey (2008). See also Results: Lateral Shift plus Repulsion.
