# Supplementary figures and images for: Sensory adaptation for timing perception
Source: Proc Biol Sci. 2015 Apr 22;282(1805):20142833. doi: 10.1098/rspb.2014.2833 (PMC4389610; doi:10.1098/rspb.2014.2833)

**A****Model**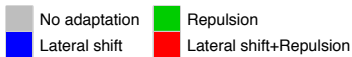**MSE**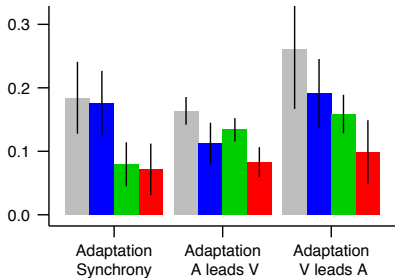**B****AIC**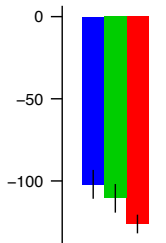

Supplement: Sup_Figure_1.pdf [file rspb20142833supp2.pdf]
